# Supplementary figures and images for: Mechanisms underlying attraction to odors in walking Drosophila
Source: PLoS Comput Biol. 2020 Mar 30;16(3):e1007718. doi: 10.1371/journal.pcbi.1007718 (PMC7105121; doi:10.1371/journal.pcbi.1007718)

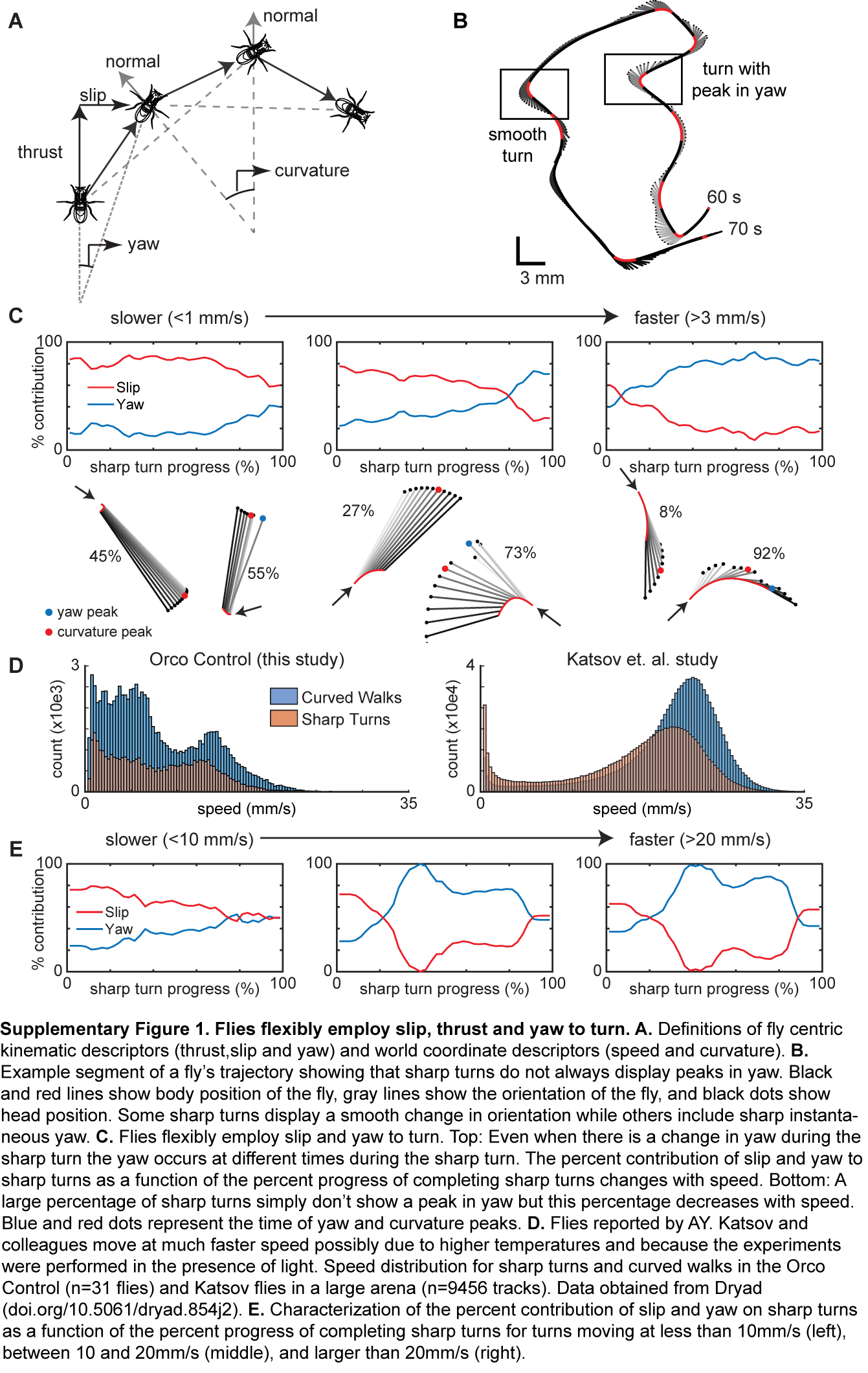

Supplement: S1 Fig — A. Definitions of fly centric kinematic descriptors (thrust, slip and yaw) and world coordinate descriptors (speed and curvature). B. Example segment of a fly’s trajectory showing that sharp turns do not always display peaks in yaw. Black and red lines show body position of the fly, gray lines show the orientation of the fly, and black dots show head position. Some sharp turns display a smooth change in orientation while others include sharp instantaneous yaw. C. Flies flexibly employ slip and yaw to turn. Top: Even when there is a change in yaw during the sharp turn the yaw occurs at different times during the sharp turn. The percent contribution of slip and yaw to sharp turns as a function of the percent progress of completing sharp turns changes with speed. Bottom: A large percentage of sharp turns simply don’t show a peak in yaw but this percentage decreases with speed. Blue and red dots represent the time of yaw and curvature peaks. D. Flies reported by AY. Katsov and colleagues move at much faster speed possibly due to higher temperatures and because the experiments were performed in the presence of light. Speed distribution for sharp turns and curved walks in the Orco Control (n = 31 flies) and Katsov flies in a large arena (n = 9456 tracks). Data obtained from Dryad (doi.org/10.5061/dryad.854j2). E. Characterization of the percent contribution of slip and yaw on sharp turns as a function of the percent progress of completing sharp turns for turns moving at less than 10mm/s (left), between 10 and 20mm/s (middle), and larger than 20mm/s (right). (TIF) [file pcbi.1007718.s001.tif]

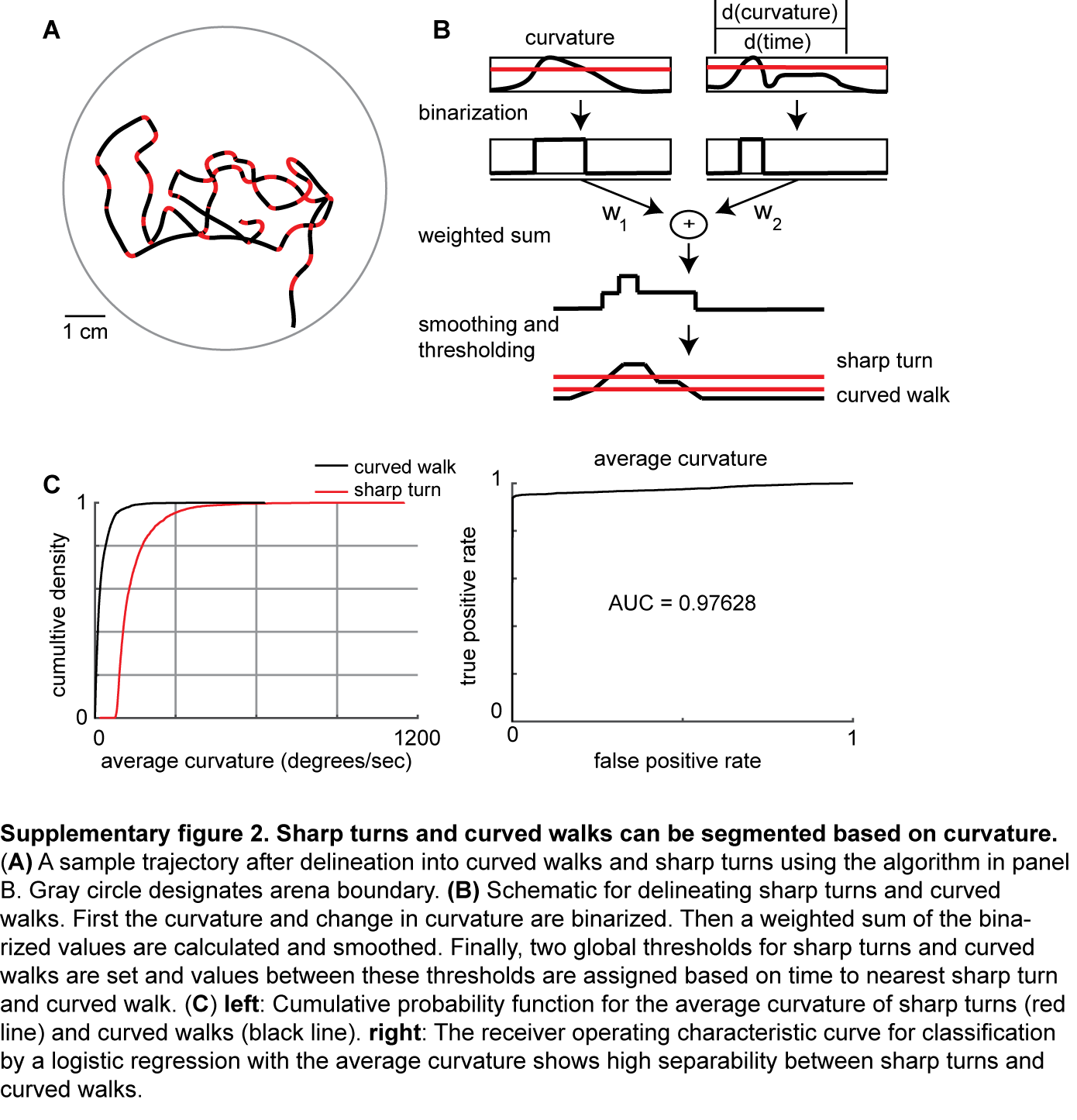

Supplement: S2 Fig — A. A sample trajectory after delineation into curved walks and sharp turns using the algorithm in panel B. Gray circle designates arena boundary. B. Schematic for delineating sharp turns and curved walks. First the curvature and change in curvature are binarized. Then a weighted sum of the binarized values are calculated and smoothed. Finally, two global thresholds for sharp turns and curved walks are set and values between these thresholds are assigned based on time to nearest sharp turn and curved walk. C left: Cumulative probability function for the average curvature of sharp turns (red line) and curved walks (black line). right: The receiver operating characteristic curve for classification by a logistic regression with the average curvature shows high separability between sharp turns and curved walks. (TIF) [file pcbi.1007718.s002.tif]

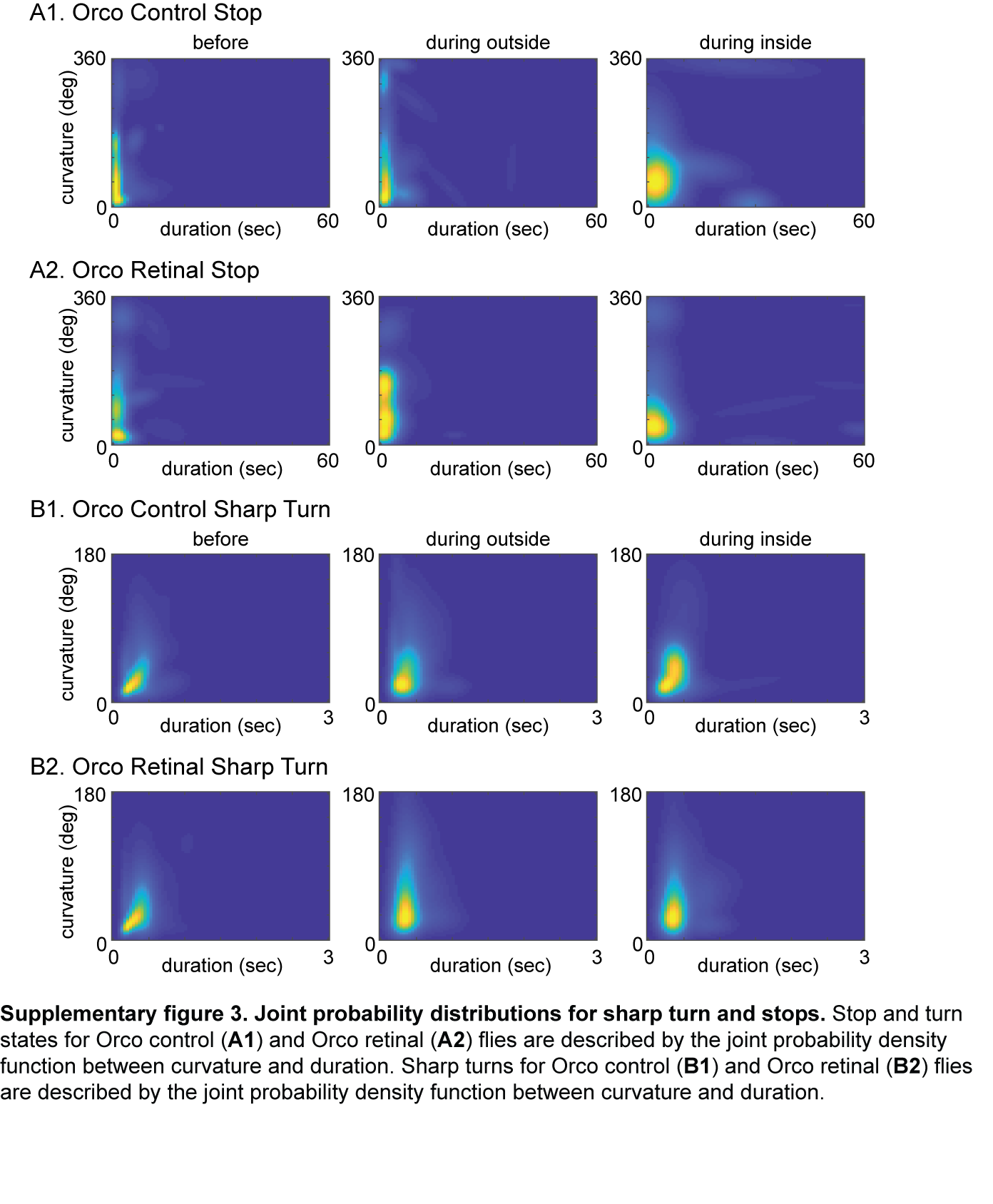

Supplement: S3 Fig — Stop and turn states for Orco control (A1) and Orco retinal (A2) flies are described by the joint probability density function between curvature and duration. Sharp turns for Orco control (B1) and Orco retinal (B2) flies are described by the joint probability density function between curvature and duration. (TIF) [file pcbi.1007718.s003.tif]

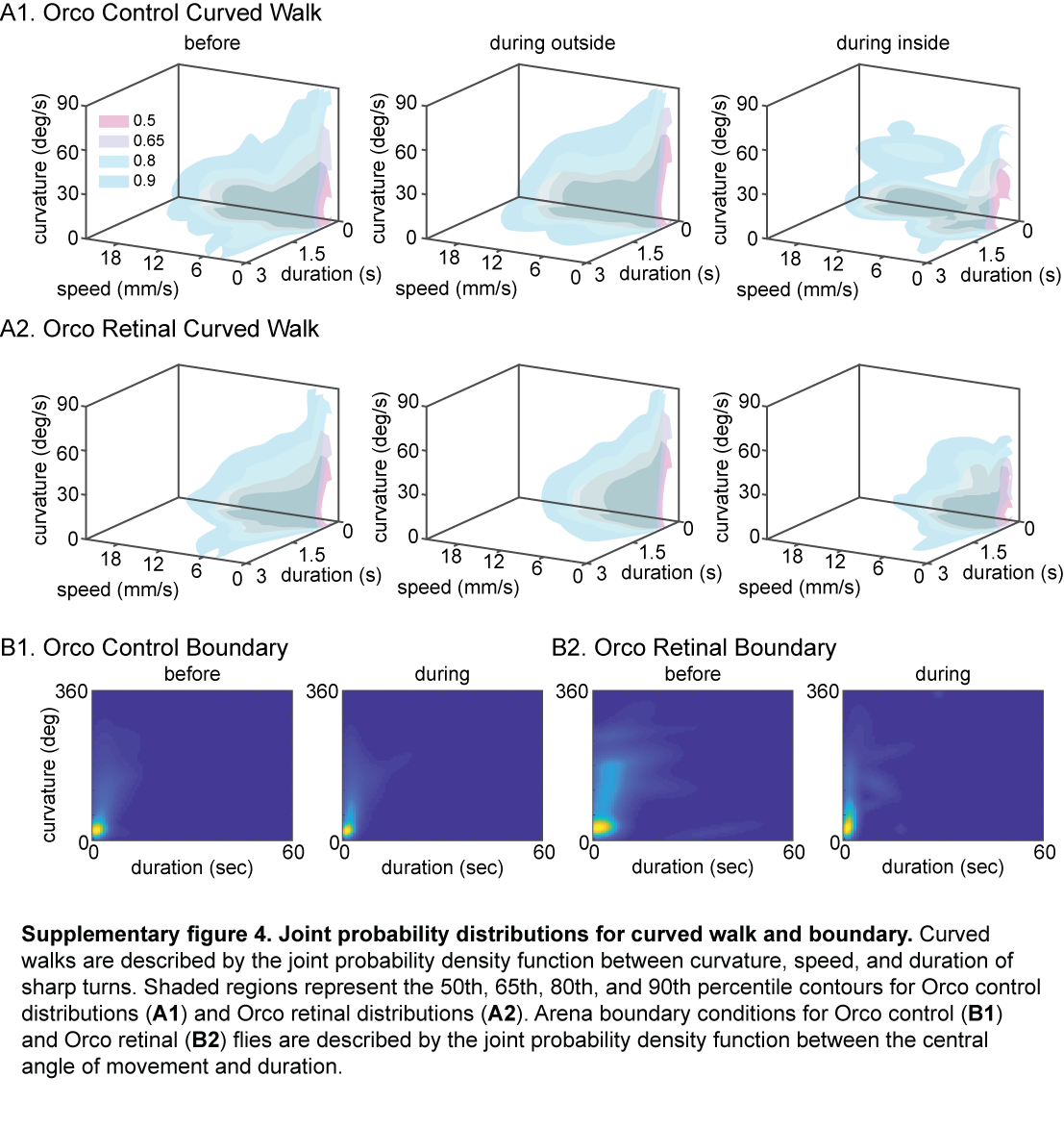

Supplement: S4 Fig — Curved walks are described by the joint probability density function between curvature, speed, and duration of sharp turns. Shaded regions represent the 50th, 65th, 80th, and 90th percentile contours for Orco control distributions (A1) and Orco retinal distributions (A2). Arena boundary conditions for Orco control (B1) and Orco retinal (B2) flies are described by the joint probability density function between the central angle of movement and duration. (TIF) [file pcbi.1007718.s004.tif]

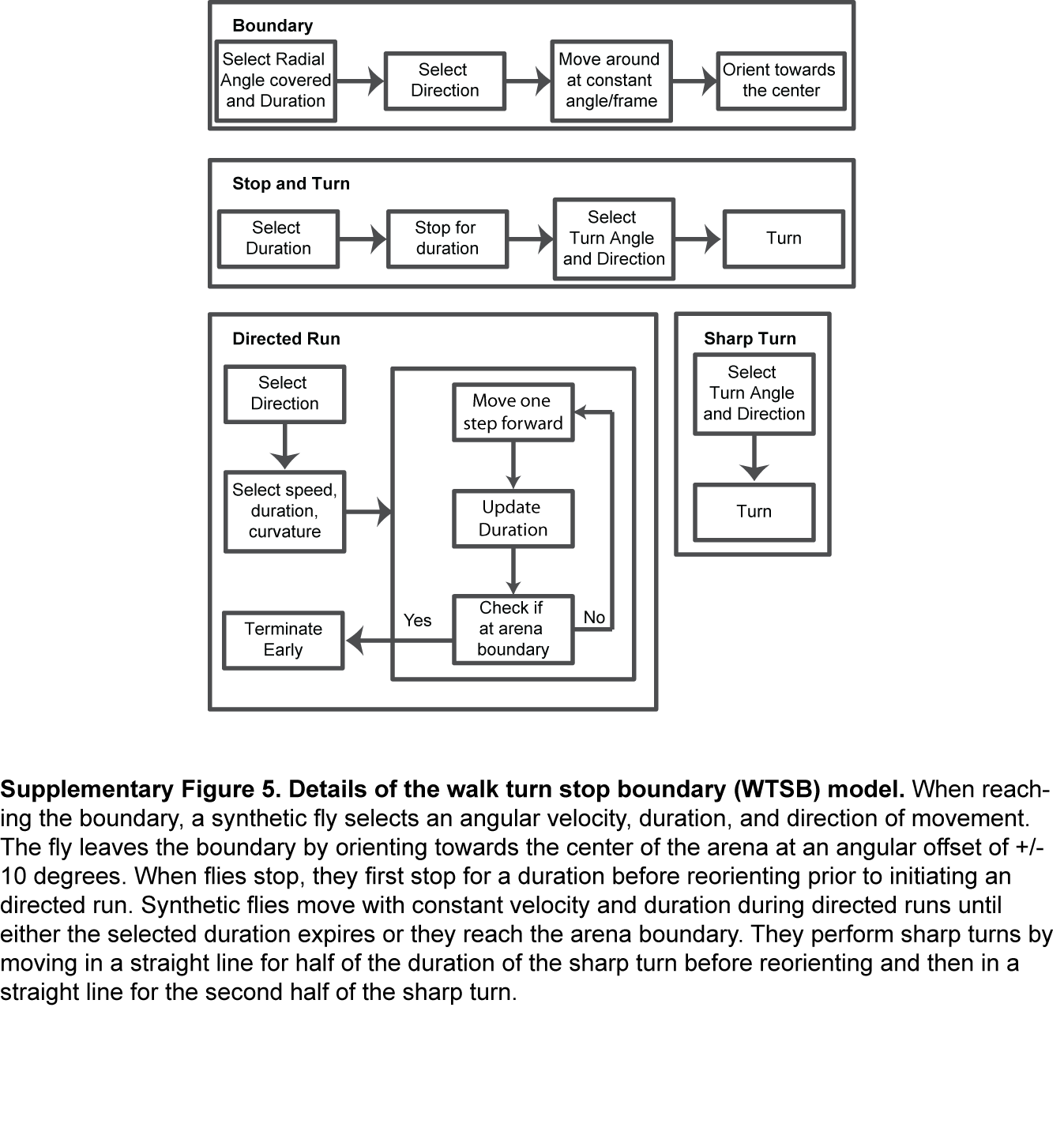

Supplement: S5 Fig — When reaching the boundary, a synthetic fly selects an angular velocity, duration, and direction of movement. The fly leaves the boundary by orienting towards the center of the arena at an angular offset of +/- 10 degrees. When flies stop, they first stop for a duration before reorienting prior to initiating an directed run. Synthetic flies move with constant velocity and duration during directed runs until either the selected duration expires or they reach the arena boundary. They perform sharp turns by moving in a straight line for half of the duration of the sharp turn before reorienting and then in a straight line for the second half of the sharp turn. (TIF) [file pcbi.1007718.s005.tif]

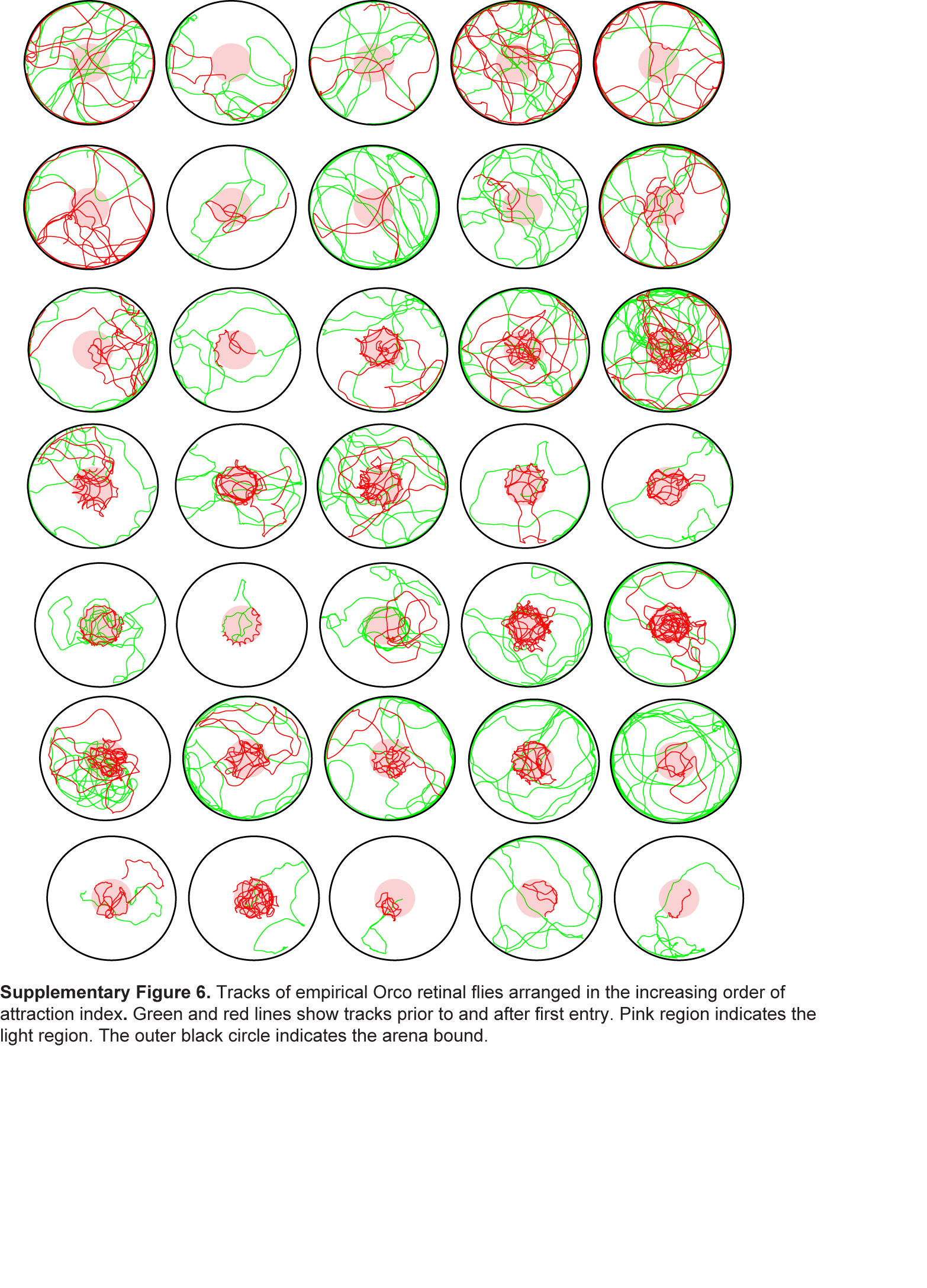

Supplement: S6 Fig — Green and red lines show tracks prior to and after first entry. Pink region indicates the light region. The outer black circle indicates the arena bound. (TIF) [file pcbi.1007718.s006.tif]

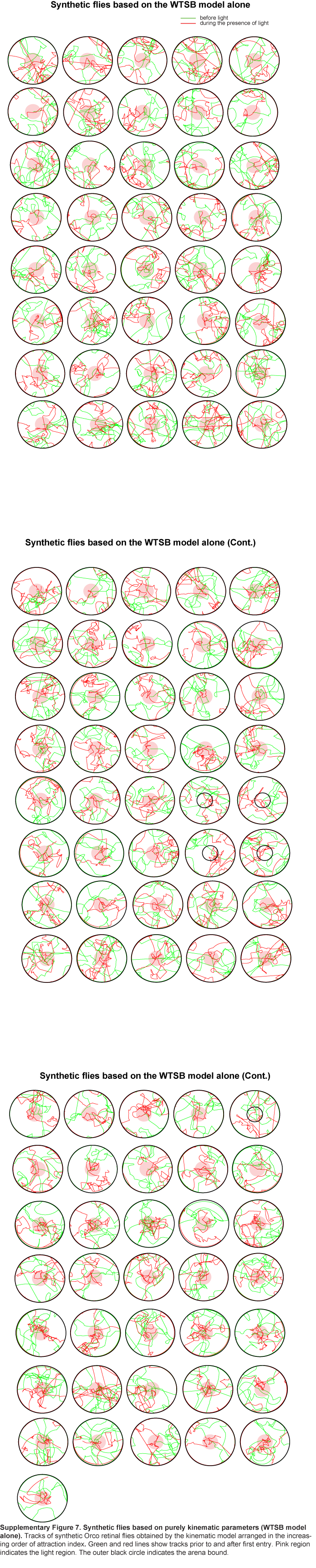

Supplement: S7 Fig — Tracks of synthetic Orco retinal flies obtained by the kinematic model arranged in the increasing order of attraction index. Green and red lines show tracks prior to and after first entry. Pink region indicates the light region. The outer black circle indicates the arena bound. (TIF) [file pcbi.1007718.s007.tif]

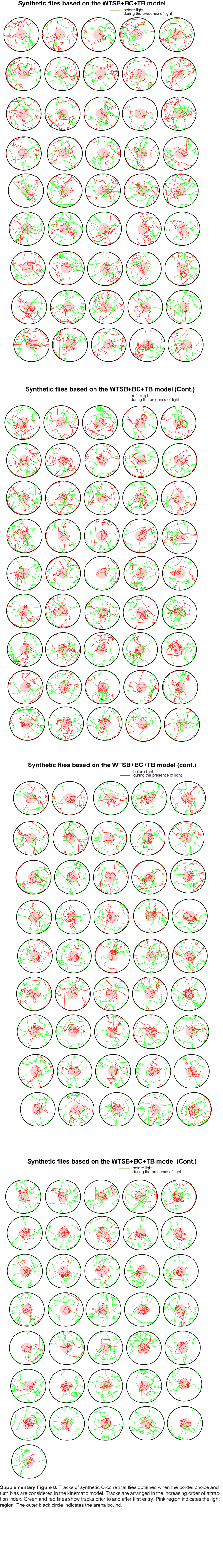

Supplement: S8 Fig — Tracks are arranged in the increasing order of attraction index. Green and red lines show tracks prior to and after first entry. Pink region indicates the light region. The outer black circle indicates the arena bound. (TIF) [file pcbi.1007718.s008.tif]

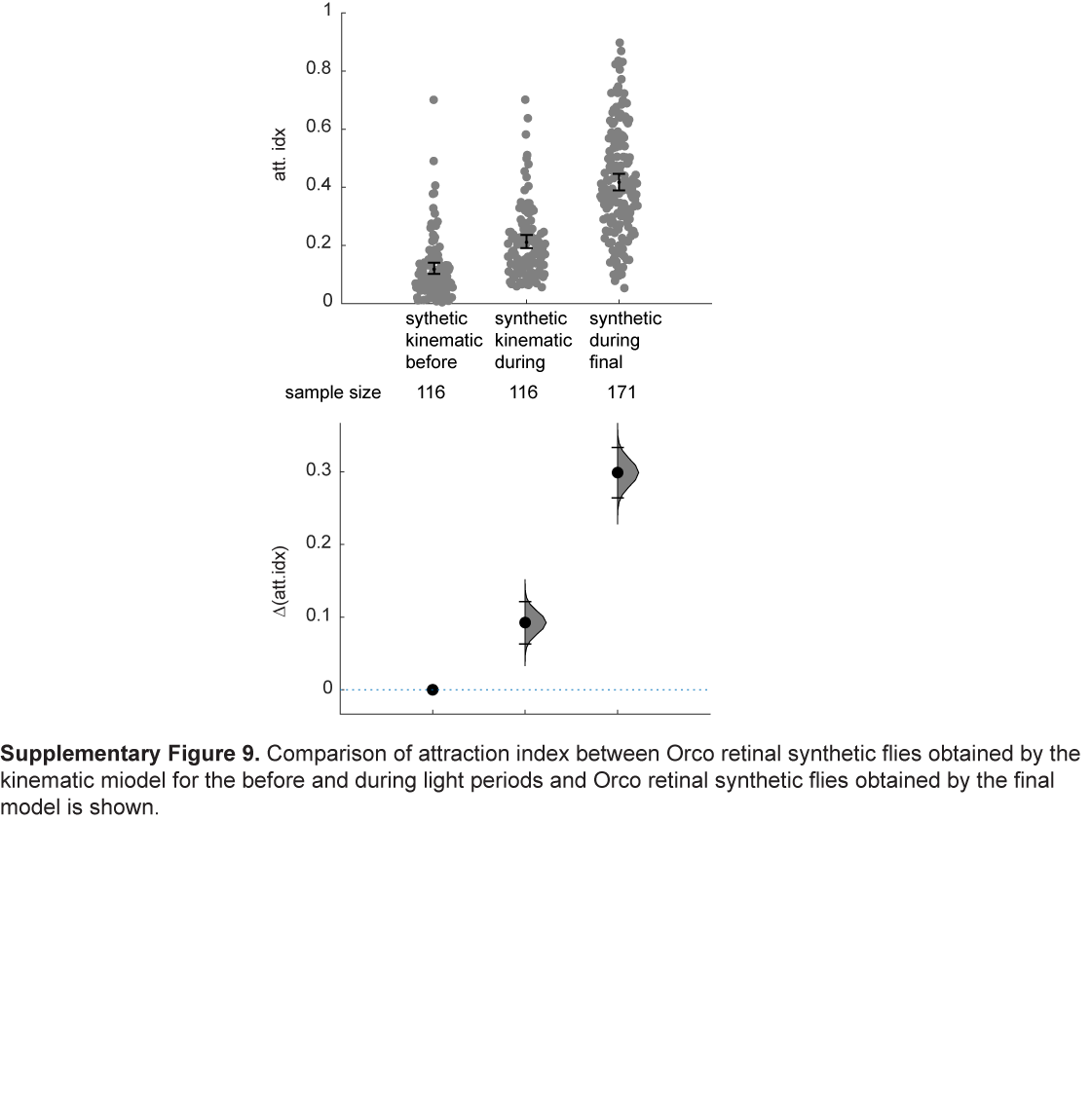

Supplement: S9 Fig — (TIF) [file pcbi.1007718.s009.tif]

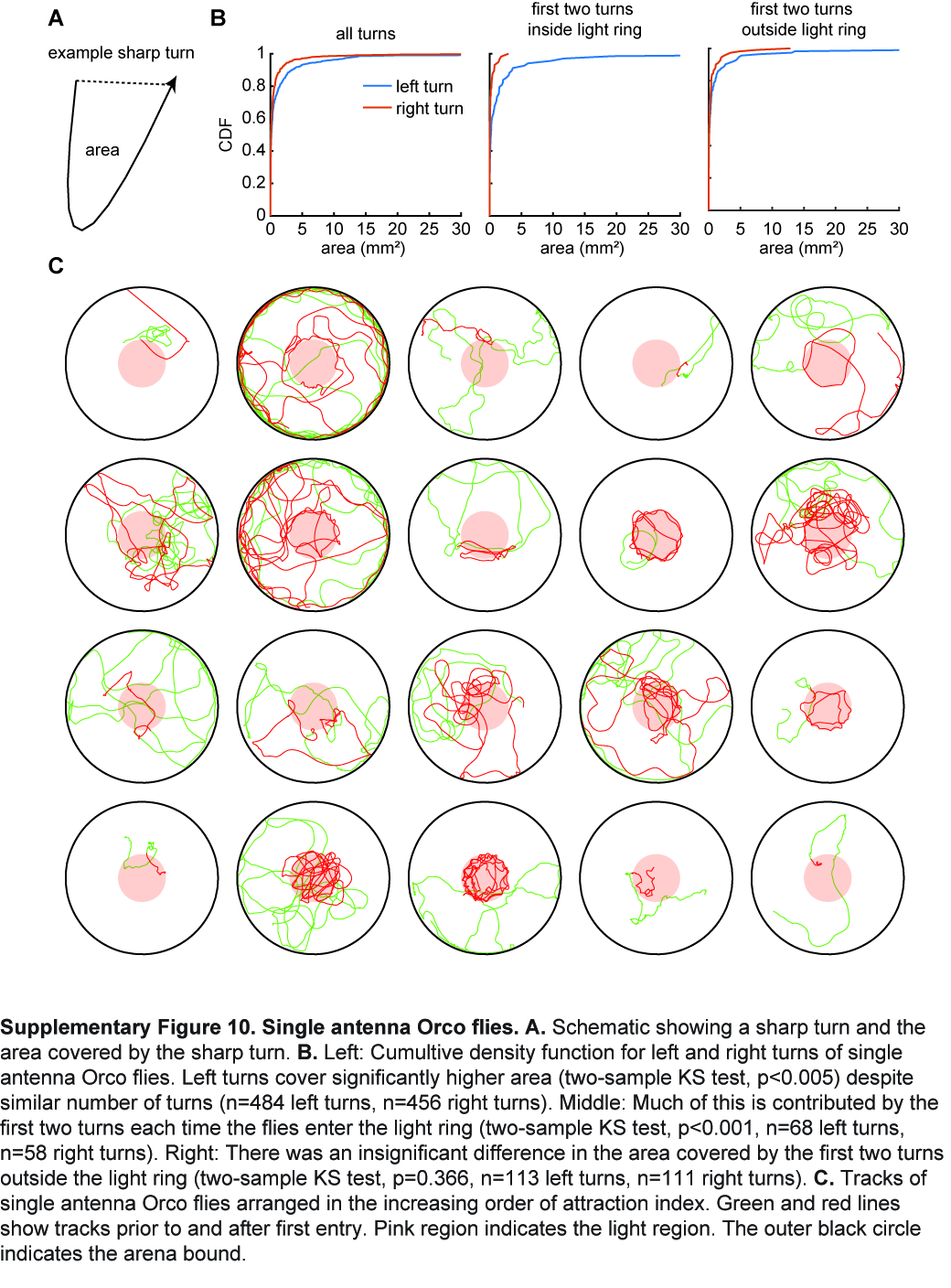

Supplement: S10 Fig — A. Schematic showing a sharp turn and the area covered by the sharp turn. B. Left: Cumulative density function for left and right turns of single antenna Orco flies. Left turns cover significantly higher area (two-sample KS test, p<0.005) despite similar number of turns (n = 484 left turns, n = 456 right turns). Middle: Much of this is contributed by the first two turns each time the flies enter the light ring (two-sample KS test, p<0.001, n = 68 left turns, n = 58 right turns). Right: There was an insignificant difference in the area covered by the first two turns outside the light ring (two-sample KS test, p = 0.366, n = 113 left turns, n = 111 right turns). C. Tracks of single antenna Orco flies arranged in the increasing order of attraction index. Green and red lines show tracks prior to and after first entry. Pink region indicates the light region. The outer black circle indicates the arena bound. (TIF) [file pcbi.1007718.s010.tif]

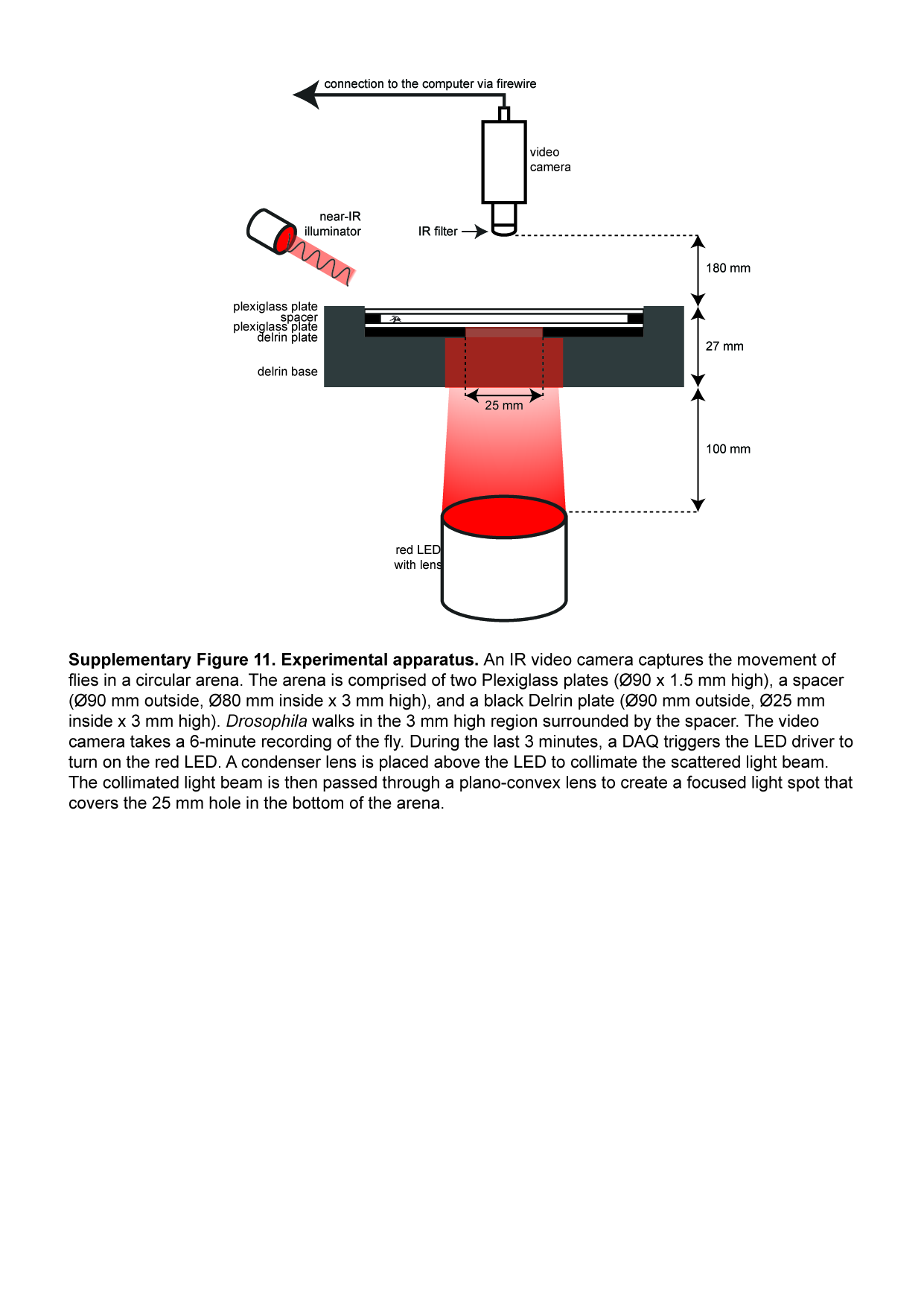

Supplement: S11 Fig — The arena is comprised of two Plexiglass plates (Ø90 x 1.5 mm high), a spacer (Ø90 mm outside, Ø80 mm inside x 3 mm high), and a black Delrin plate (Ø90 mm outside, Ø25 mm inside X 3 mm high). Drosophila walks in the 3 mm high region surrounded by the spacer. The video camera takes a 6-minute recording of the fly. During the last 3 minutes, a DAQ triggers the LED driver to walks in the 3 mm high region surrounded by the spacer. A condenser lens is placed above the LED to collimate the scattered light beam. The collimated light beam is then passed through a plano-convex lens to create a focused light spot that covers the 25 mm hole in the bottom of the arena. (TIF) [file pcbi.1007718.s011.tif]
