# Supplementary material for: Mechanisms underlying attraction to odors in walking Drosophila
Source: PLoS Comput Biol. 2020 Mar 30;16(3):e1007718. doi: 10.1371/journal.pcbi.1007718 (PMC7105121; doi:10.1371/journal.pcbi.1007718)
Supplement: Attachment — Submitted filename: point-by-point response_v2.pdf [file pcbi.1007718.s013.pdf]

Point-by-point response

Reviewer 1:

Were the experimental flies male, female or a mix of both?

We only employed female flies as is traditional for Drosophila olfactory experiments. We have now explicitly stated the same (L8 in Methods)

Please make clear that only retinal-fed flies will express the Chrimson channel.

We have clarified that only the retinal-fed flies will have active Chrimson channels (L95 in the main manuscript).

Were flies housed in the dark prior to experimentation? Fluorescent lights may contain enough red light to activate the Chrimson channel.

Yes they were. We have specified this in the methods (L9).

It is my understanding that Drosophila and other insects cannot see red light. This seems like a crucial point to make, since flies can exhibit phototactic behavior, and this could be an alternative mechanism to explain their attraction to the optogenetic stimulus.

Great point! We have made this clear (L84-85 in the main manuscript).

I'm assuming the wings were removed from the flies prior to the experiment. Please make this point clear.

No, the wings are not removed. The flies are forced to walk because they are between two plexiglass plates. The reviewer's comment made us realize that a schematic of the behavioral apparatus would be useful. A schematic of the apparatus is now included as Supplementary Figure 11.

I'm assuming the Orco-Gal4 gene has been shown to be encoded in ORN's; and furthermore, that it has been shown to encode something attractive to flies. It would be useful to know what the odorant is (if it is known).

We have now included the details on Orco-Gal4 (Line 91).

It would be useful for readers to know that fly ORNs are located on the antennae. Therefore, removing an antennae would be reducing a bilateral system to a unilateral one.

Thanks! This information is included in the figure legend.

In prior work, the same authors concluded that individual flies' behaviors in a similar task cannot be represented by the behavior of an average fly, but were separable into several distinct clusters based on a 10 state Hidden Markov Model. Compared to the authors' prior work, the WTSB model seems to be limited in scope with less utility. Perhaps the authors could make some predictions about untested behaviors using the WTSB?

The Hierarchical Hidden Markov Model (Tao et. al. 2019) serves a different purpose compared to the WTSB model. There are two big differences: 1) The WTSB model is designed to capture the fly's distribution in the arena, whereas the HHMM model describes the speed-curvature timeseries. By modeling the boundary condition, we are able to describe the distribution of the fly in the arena. 2) The HHMM model is rigid in the sense that it is a kinematic model and it is hard to tune the model to capture

features of the data, it is particularly difficult to model non-orienting strategies using the HHMM. The WTSB model is flexible, and we could model border choice and turn bias (Figure 6) to model orienting strategies. We had covered this point in the last paragraph of the discussion. We have further elaborated on this point in the revised manuscript.

These two sentences in the abstract seem contradictory. "Second, the fly turns more at the border between light-zone and no-light zone and these turns have an inward bias. Surprisingly, there is no increase in turn rate, rather a large decrease in speed that makes it appear that the flies are turning at the border." How can the fly turn more, but not increase its turn rate? This result would make me double-check my methods for identifying turns.

The flies turn more simply because they spend more time at the border. We have rewritten that part of the abstract to make it clearer.

The authors repeatedly refer to fly "kinematics." Encoding of kinematics (motion of muscles) vs kinetics (forces necessary to produce movements) is a debate established in motor neuroscience. Not sure you want to use this jargon here, as it applies to fly neurobiology.

This is a good point. We thought hard about it. Ultimately, we have decided to stick to kinematics because that word is really appropriate here. Kinematics and kinetics are such common words and used in the context of anything that moves, we hope that the reader will not immediately associate it with limb kinematics and look to figure out whose movement we are trying to describe. I hope that the reviewer understands our viewpoint.

In the introduction, the description of "navigational movements" may be better described as "goal directed movements" or "orienting movements towards a stimulus."

We respectfully disagree with the reviewer. "Goal directed" can imply things like reaching movements or memory-related tasks. "orienting movements towards a stimulus" does not include processes like path integration. Based on a quick search we found that the phrase "navigational movement" does appear in the literature so we hope the reviewer is all right with us not making the switch.

The authors make a point that path integration is not necessary to describe behavior in their models. However, I feel that the experiments are somewhat limited in their ability to disentangle path integration from "kinematic" models of decision-making in the fly as the appetitive stimulus is relatively large compared to the size of the arena. Path integration may come into play when either a) the appetitive stimulus is small compared to the arena size; or b) the arena size is large with multiple available stimuli to explore.

We are not saying that path integration does not happen or cannot happen in a more demanding scenario. We are just saying that without taking into account changes in speed, and sharpness of turn, any claims of path integration is premature.

Results "ORN activation alone in the absence of air can mediate robust attraction." I believe the authors meant to say in the "absence of wind" or in the "absence of air currents." However, note that in even relatively still air in the laboratory, there will still be some air currents. It may be worth measuring and reporting these with an anemometer, since flies can exhibit anemotaxis.

Thanks! We have used the same phrase we used in the main text – "absence of directional wind cue" (Line 75)

It is not clear to me how the authors parse out "stops." I'm assuming it's when velocity is below some threshold.

Yes. It is reported in the methods: "When the fly's speed was less than 0.5 mm/s, we assigned the fly as being in the stop state."

Line 203 states that "the synthetic flies are not as attracted to the light-zone as the empirical flies." Why would they be? The model has no mechanism to respond to rewarding stimuli.

Kinematic changes are an important mechanism for attraction. As detailed in the introduction, often kinematic changes are the only mechanism for attraction. Furthermore just kinematic changes do mediate small but statistically significant attraction (See Figure S9).

Line 255 A deeper analysis of the effect of removing an antennae seems to be warranted. The flies appear to spin preferentially in one direction. This suggests to me that the flies were using a binaral or stereo-olfaction mechanism for detecting the edge of the stimulus.

We have performed a more detailed analysis of the antenna-less flies. The antenna-less flies don't show a preference in turn direction – right turns are as common as left turns. However, we did find one interesting difference between normal retinal fed flies and flies with just one antennae: inside the odor-zone, the antennae-less flies take right turns that are more loopy than the left turns (Supplementary Figure 10).

Line 266 "The increased turning at the border is due to the decrease in speed as the fly exits the light-zone." Is this consistent with klinotaxis or not?

Strictly speaking, it is not because an increased turn frequency is necessary.

Line 292 "It is clear from our data that flies not only select the duration of each walk, but also its speed and curvature." Please clarify.

Unlike previous in which walks were modeled with the same speed and no curvature, we show here that flies walk at different speeds and curvature on each walk. We have added one more sentence to clarify this point further (L 315).

Line 342 "Essentially, a circuit that relates decreasing ORN response to decreased walking speed and increased turn angle during sharp turn is all that is necessary to produce robust attraction to a stimulus." This seems like a key point. Can you propose such a circuit or how it might be implemented by Drosophila neurobiology?

We can't point to specific neurons that might be performing this computation. The point we were trying to make here is that a simple circuit can perform this computation.

The Hungarian algorithm approach to determining head position seems novel and may warrant further explanation. Otherwise, please provide a citation where this method has been used before.

The use of Hungarian algorithm in this case is just to assign the head and tail positions. The algorithm efficiently minimizing the total distance moved by all points of interest between two consecutive frames. This method would be more useful as the number of interested points increase (perhaps simultaneously tracking more flies). We have added this description to the methods.

1-1.3s LOESS smoothing seems like oversmoothing to me. I would be more comfortable if a uniform smoothing was used throughout.

We understand the concern. The reason for the smoothing was to prevent overfitting in the delineation of sharp turns and curved walks and because we were interested in overall movement paths and not the small fluctuations along the path that may be caused in part by tracking or just fluctuations in movement steps. We concede that there is likely a better method than the one used in this study, but we have made sure that differences between unsmoothed and smooth tracks were less than 0.0037 mm for each time point.

Speed is given as both variables d and s in the methods section.

Thank you for pointing this out. We have now changed all instances of speed to *s*.

I am confused as to the difference in Figure 4D and Figure 5G. This appears to be the same data, but the result is different.

We understand the confusion. Figure 4 and 5 have to do with different models. Figure 4 is just the WTSB model. Parts of the former Figure 5 (now Figure 6) is a different model in which border choice and turn bias were incorporated. We have made several changes to make this difference between the two models clearer.

Figure 5B, is it noteworthy that the peak of turn density is near the inflection point of the laser intensity?

Yes it is. We have noted the same more prominently.

Supplementary Figure 1 (right panel). Did you analyze data from the Katsov, et al. study? If so, please state where this data is available or if you got it through contact with the authors.

Thanks. We have now done this in the figure legend.

Supplementary Figure 2B. Please include the y-axis and thresholds in the plots of curvature and derivative of curvature.

Supplementary Figure 2B is just a schematic of the process. As such, the axis value and threshold are unnecessary. The thresholds (best-fit parameters) used in this study are listed in table form at the end of the methods section labeled "Segmentation of tracks into the four states: boundary, walk, sharp turns and stop"

Supplementary Figure 3. What is the significance of including this figure?

It shows the empirical distribution.

Supplementary Figure 4 (caption). What do the authors mean by the "central angle of movement"?

The difference in the angular coordinate in polar space entering the boundary location and the angular coordinate in polar space leaving the boundary location.

Supplementary Figure 6-8. Compliments to the authors on displaying all the raw data.

Thank you! We have now also displayed the unilateral antenna flies in Figure S10.

Reviewer #2:

Multiple studies have explored how odors are represented in the peripheral olfactory system of Drosophila and how freely-moving flies respond to odor plumes carried by turbulent air flows. In spite of this work, very little is known about the orientation behavior of flies in response to pure olfactory stimulations. Bhandawat and colleagues fill this important gap by using an elegant experimental paradigm. They take advantage of optogenetics to activate all olfactory receptor neurons (ORNs) in the central region of a circular arena. This effectively creates a binary odorant landscape where flies accumulate in the light zone (or odor zone). Using state-of-the-art behavioral analysis, the authors quantify how odor stimulation modifies the kinematic parameters of the fly locomotion.

To ask whether changes in kinematic parameters are sufficient to reproduce the observed behavior of real flies, the authors turn to agent-based modeling. The simulations point out the insufficiency of a purely kinematic model of foraging. Further analysis bring the authors to establish that a crucial component of the fly's navigation algorithm is the ability to reorient toward the source upon exiting the odor zone. Surprisingly, the authors report that reorientation does not involve a bias of turns toward the gradient but that it arises from a deceleration of the fly followed by sharp turning. Given the idiosyncratic nature of the assay (the all-or-none nature of the light landscape and pan-neuronal activation of the peripheral olfactory system), it is unclear whether this conclusion can be generalized to more natural conditions of olfactory stimulations.

Overall, the research is well designed and neatly executed. The manuscript is clearly written (in particular, the introduction abounds with useful references). The conclusions are interesting and of general interest.

Thank you for the kind words on our manuscript. We are also sympathetic to the critique about the all-or-none nature of the light landscape and activation of many ORN classes (it is not pan-neuronal, however). We are actively working on both issues. Having said that and recognizing that in some scenario there might be an odor gradient, animals navigating over long distances face a turbulent environment. In this environment, animals do encounter odors in all-or-nothing bursts (see for example : Murlis, John, Mark A. Willis, and Ring T. Cardé. "Spatial and temporal structures of pheromone plumes in fields and forests." *Physiological entomology* 25.3 (2000): 211-222). Essentially, providing different spatio-temporal pattern of all-or-none stimulus is closer to the naturalistic stimulus encountered by animals such as flying insects. This manuscript is a first step to understanding this naturalistic pattern.

Major comments/suggestions:

1. In lines 113-114, the authors make a case that "even the straight walks are curved". This statement is supported by the trajectory segments shown in Fig 2. But it also begs the question of why many trajectories in Supplementary Fig. 7 include long segments that are perfectly linear. These straight segments appear to be inexistent in Supplementary Fig. 6. Could these straight segments in Supp. Fig 7 be due to a tracking artifacts? If not, the authors should look into the behavioral significance of segments devoid of any curvature.

Excellent point! Supplementary figure 6 shows the empirical trajectories of real flies. These support our claim that even the straight walks are curved. However, when using the fitted probability distribution functions (pdfs) for curved walks, it is possible to get trajectories with no curvature (See Supplementary Figure 4a) because there is a small probability of zero curvature. This is reflected in the synthetic fly trajectories shown in Supplementary Figure 7, where the curved walks are sampled from the distributions shown in Supplementary Figure 4.

We have also looked in much closer detail at our synthetic trajectories and found that there are some instances where the fly jumps from one boundary location to another, which when visualized looks like a long linear segment. This was because the boundary condition joint pdf between angle of movement and duration allows for the ability to have a short (1-2 frame) duration with a large (30+) angle of movement. Both issues unfortunately arise due to the difficulty in accounting for hard boundary conditions in estimating pdfs.

To test that these inconsistencies do not affect the result, we imposed a hard 10 frame (~330 ms minimum) cut-off for the duration of boundary conditions and a small (0.0001 deg/s) curvature minimum for curved walk curvatures. Running models with these hard boundary conditions showed the same summary results as the model shown in this paper without the long straight tracks.

2. In Figs. 4 and 5, the authors use their model to make a convincing case that a non-reorienting model (WTSB) is insufficient to account for the behavior of real flies stimulated by an odor gradient. They go on to show that the introduction of a "border choice" and a "turn-bias" parameter can account for the ability of flies to re-enter the odor zone upon its exit. This is done by training and "\validating the model on the same dataset. To validate the model further, I would encourage the authors to monitor the behavior of Orco-Gal4;UAS-Chrimson flies in a gradient with a geometry different from the binary landscape - for instance a Gaussian gradient produced by a filter. Testing the behavior in a smoother light gradient might reveal whether flies are capable of biasing their turns toward the source when the stimulus landscape is not binary.

In the paragraph that starts on line 340, the authors argue that reorientation maneuvers based on slowing down followed by (non-biased) sharp turning could account for the navigation of walking flies. Although this conclusion is plausible, it would be strengthened by a validation of the model in more realistic olfactory landscapes than that on which the model was trained.

This is an excellent idea, but it is beyond the scope of this particular study. As argued earlier in response to the reviewer's overall assessment, most of the time, animals encounter odors in

bursts of varied duration and inter-burst intervals. Thus, we are not strongly motivated to analyze the odor gradient condition. The central claim in this study is that 1) kinematic change, 2) orientational cues, 3) memory-based mechanisms must all be investigated to understand an animal's strategy. The exact strategy employed will depend on the stimulus profile. Previous studies have largely ignored kinematic changes.

3. Fig 5B shows that more turns take place inside the odor zone. This point is also evident from trajectories shown in the main figures. Yet, the authors did not establish the ability of their model to reproduce the behavior of flies inside the odor zone. How do flies behave when they experience an all-or-none increase in ORN activity while re-entering the odor zone? Although one might predict that flies move straight as soon as they reenter the odor zone (a surge response that would be compatible with the model of Alvarez-Salvado & Nagel 2018), real flies appear to behave differently. Is the turning dynamics inside the odor zone the same as that outside the odor zone (excluding the interface)?

Excellent point. We show that the model does replicate the behavior of the flies. To address this critique, we have split Figure 5 into Figures 5 and 6. We have also included a panel showing that the model behaves just like the empirical flies in terms of their propensity to turn at the border (Figure 6B).

4. Do flies behave differently upon optogenetic stimulation of all ORNs compared to when stimulated by real odors as was studied by Jung & Bhandawat in 2015?

The Jung and Bhandawat manuscript showed that different odors produce changes in different kinematic parameters so it is hard to compare. If we compare to apple cider vinegar, there are some similarities and some dissimilarities. We have discussed this in detail (Lines 335-350 in discussion).

5. In line 265, the authors refer to behavior "in the absence of light". Do they mean "in the absence of retinal"? Although this difference might look purely semantic, it is associated with a potentially more profound problem. The authors have not ruled out that flies respond to abrupt changes in red light through their visual and/or thermosensory system. Although flies are not supposed to orient to red light, It would be important to exclude that the all-or-none red stimulation does not induce startles. The authors could conduct this control by extending their analysis to a condition where flies do not experience any light change. In behavior devoid of light stimulation, they could apply a behavioral analysis with a "mocked odor zone" and quantify the change in kinematic parameters when flies exit this "mocked zone". This no-light control would nicely complement the no-retinal control.

Thank you! The reviewer is correct in that particular case, and we have corrected the same. In general, we do have a "mock odor-zone" because all experiments are performed with a 3-minute control period during which there is no light.

5. Could the authors take advantage of their orienting model to account for differences in the behavior observed between unilateral and bilateral sensing in Fig. 6A2 and 6A2? How different would the overall reorientation dynamics be if flies were capable of biasing their turns toward the source (Fig. 6C1)? The agent-based model appears to be ideal to address this question. In their discussion of the results observed with flies having unilateral olfactory function, the authors should refer to previous work by Frye (Duistermars & Frye, CB 2009) and Nagel (Alvarez-Salvado & Nagel, eLife 18).

We have cited the references suggested here. Thanks for the suggestion! The reviewer's suggestion of adding a bias to see what would happen is a good one. However, that analysis has already been done by *Alvarez-Salvado & Nagel, eLife* study. They found that if they add a small bias there is not much change; a large bias makes a difference. We will likely observe the same result.

Minor comments/suggestions:

- Abstract: The authors state that they "create a new behavior". Stating that they created a new conditions of olfactory stimulations might be more appropriate.

Good point. Thanks! We took this suggestion.

- The kinematic analysis is based on the average behavior of multiple flies. Recent work by the lab of de Bivort and the authors has highlighted the high degree of variability in olfactory behaviors across individual flies. Does this inter-individual variability partly invalidate the development of a model based on behavioral averages? Whereas the dataset obtained for individual flies might not be sufficient to build and validate agent-based models, this issue should be addressed in the manuscript. Wouldn't the HHMM framework previously proposed by the authors be perfectly suited for this analysis?

We had touched upon this point in the previous submission, but we have now expanded on this point. See last paragraph of the discussion. Essentially, the reviewer is correct that individuality limits the development of a model based on the average fly. But this model also has an advantage over HHMM in that it is flexible. It would be difficult to model border choice and turn bias using the HHMM model. We will explore the issue of individuality with the WTSB model at a later date when we have a larger dataset with the same fly interrogated over many trials.

- Classifiers are designed to distinguish curved walks from sharps turns. In Supplementary Fig. 2, the performance of these classifiers is evaluated by conducting a ROC analysis. Presumably, the true and false positives were defined based on a ground truth. However, I didn't find in the text a description of what the ground truth corresponds to. This point should be clarified.

In this case, we are asking if there is clear separability between sharp turns and curved walks that were classified from our optimization method. As such, in this case, the sharp turns and curved walks were our “ground truth”, and we used a binary logistic regression classifier based on the average curvature of curved walk and sharp turn tracks.

- In Fig. 3A, the authors compare real trajectory segments with synthetic trajectory segments simulated with the kinematic model. How was this comparison achieved? Did the authors make the synthetic fly start with the same initial conditions as the real fly to obtain the segments shown in Fig 3A? If so, how did they select the synthetic segments shown in the comparison with the experimental segments? Additional details related to this comparison between experimental and simulated behaviors should be provided in the figure caption and the main text.

All synthetic flies were initialized to start at the exact center of the arena facing to the right. As the model assumes that the fly is walking in an unit circular arena (normalized) centered at (0,0), this means that synthetic flies begin at (0,0), with an initial heading direction of 0 degrees. Synthetic flies were selected based on whether or not the flies

1.) Reached a distance of at least 1.1x the radius of the light boundary outside and 0.9x the light boundary inside in both the before and during scenarios (which is consistent with our experimental criterium)
2.) Had a first entry time within the 85th percentile of that of the empirical flies.

We have updated the methods to make these points more clear.
All tracks are shown in the supplementary figures. The specific tracks shown in the main figures are representative tracks.

- In Fig. 5C1, the blue and black dots are difficult to tell apart.

Thank you for noticing. We have changed the color of the blue dots to a lighter more turquoise color.

- In Fig 5D2, the "first-two-turns" and "later-turn" probability function is defined for radial distance inside the odor zone. Does this mean that the turn bias toward the source is calculated for positions inside the odor zone? Wouldn't this be inconsistent with the definition of the inward turn, which assumes the the position is outside the zone?

The turn in bias means turning towards the absolute central location of the circular arena, not just the light region. Since the light ring is concentric to the arena, when the fly is outside of the light region, the turn in bias means turning towards the source of the light stimulus. When the fly is inside, the turn in bias means not wanting to turn away/leave from the source of the light.

- Line 296: I didn't understand why each state must necessarily last 1 second. Could this be further explained in the main text?

Good point. We meant 1 second on average.

Typos:

- Line 45: "random walks has been" -> "random walks have been"

Thanks! Corrected.

- Line 142: To be consistent with the figure labeling and the main text, 2A2 should be written with 2 as a subscript.

Thanks! Changed.

- Line 172: The parenthesis including Figure S3 and Figure S4 should be closed.

Thanks! Changed.
