# Supplementary material for: Mechanisms underlying attraction to odors in walking Drosophila
Source: PLoS Comput Biol. 2020 Mar 30;16(3):e1007718. doi: 10.1371/journal.pcbi.1007718 (PMC7105121; doi:10.1371/journal.pcbi.1007718)
Supplement: Attachment — Submitted filename: StraightWalks.pdf [file pcbi.1007718.s014.pdf]

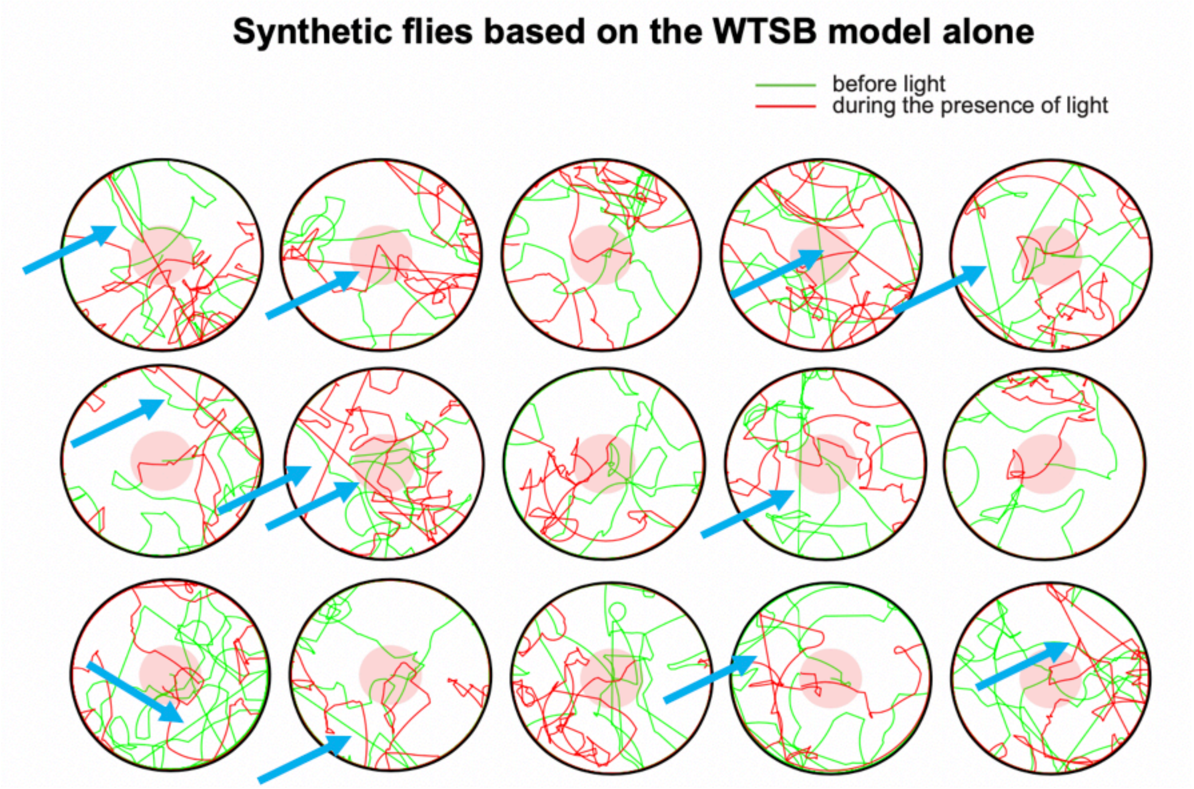
Synthetic flies based on the WTSB model alone
before light
during the presence of light
